# Post-COVID-19 *Pneumocystis* pneumonia cases from Pakistan: an observational study

**DOI:** 10.1099/acmi.0.000406

**Published:** 2023-01-05

**Authors:** Hammad Niamatullah, Nosheen Nasir, Kauser Jabeen, Salima Rattani, Joveria Farooqi, Najia Ghanchi, Muhammad Irfan

**Affiliations:** ^1^​ Research Facilitation Office, Aga Khan University, Karachi, Pakistan; ^2^​ Department of Medicine, Aga Khan University, Karachi, Pakistan; ^3^​ Department of Pathology and Laboratory Medicine, Aga Khan University, Karachi, Pakistan; ^4^​ Department of Pathology and Laboratory Medicine, Aga Khan University, Karachi, Pakistan; ^5^​ Department of Pathology and Laboratory Medicine, Aga Khan University, Karachi, Pakistan; ^6^​ Department of Pathology and Laboratory Medicine, Aga Khan University, Karachi, Pakistan; ^7^​ Department of Medicine, Aga Khan University, Karachi, Pakistan

**Keywords:** COVID-19, fungal infection, Pakistan, *Pneumocystis* pneumonia, superinfection

## Abstract

**Background**. Concurrent coronavirus disease 2019 (COVID-19) and *Pneumocystis jirovecii* pneumonia (PJP) has been described in various reports, with a recent study describing a 9.3 % *P*. *jirovecii* detection rate in critically ill COVID-19 patients.

**Methods**. Patients with PCR-confirmed PJP following COVID-19 infection who were admitted to Aga Khan University Hospital, Karachi, Pakistan from March 2020–June 2021 were identified through a laboratory database. Detection of severe acute respiratory syndrome coronavirus 2 (SARS-CoV-2) virus was performed by RT-PCR Cobas SARS-CoV-2 qualitative assay. *P. jirovecii* PCR was performed using the RealStar *Pneumocystis jirovecii* PCR kit. Clinical, radiological and laboratory data for PJP patients were recorded.

**Results**. During the study period, 3707 patients were admitted with COVID-19 at our hospital. *P. jirovecii* PCR was requested for 90 patients and was positive in 10 (11 %). Five out of 10 patients were discharged from the hospital and later developed cough and dyspnoea. Five patients remained hospitalized with severe COVID-19 and developed PJP. Eight patients in our study received systemic steroids. The trends of lymphocyte counts of all patients showed a lymphocyte count of <1000 mm^−3^ (<1.0×10^6^ cells µl^−1^) in the week of PJP diagnosis. Four patients did not survive; one of these patients did not receive co-trimoxazole due to late diagnosis, one patient had concomitant nosocomial pneumonia and bacteraemia with multidrug-resistant *Acinetobacter* species, and two patients had concomitant aspergillosis.

**Conclusion**. In summary, invasive fungal infections such as PJP should be considered as a complication in COVID-19 patients, with prompt evaluation and management.

## Introduction

Infection with severe acute respiratory syndrome coronavirus 2 (SARS-CoV-2) leads to severe illness in ~20 % of patients, requiring hospitalization [1 ]. Some of these patients develop a critical illness with respiratory failure, acute respiratory distress syndrome and multi-organ system dysfunction [1]. It is known that after viral infections patients are more prone to fungal infections; this has also been seen in patients affected by coronavirus disease 2019 (COVID-19) [2 ]. This occurs because of generalized immune system dysregulation and damage to the respiratory epithelium. SARS-CoV-2 seems to specifically affect cell-mediated immunity, as the absolute counts of both CD4^+^ and CD8^+^ T-lymphocytes are significantly lower in patients with severe COVID-19 [3 ].

*Pneumocystis jirovecii* is a fungal organism that is a colonizer of the human respiratory tract, but causes opportunistic pneumonia in immunocompromised patients. It disproportionately affects immunocompromised individuals, especially those affected by human immunodeficiency virus (HIV) with a CD4 count of <200 cells µl^−1^. Other conditions that predispose to *P. jirovecii* pneumonia (PJP) are malignancies, solid organ transplant and chronic inflammatory conditions, especially those that require the use of systemic steroids or immunosuppressive agents [4 ]. Many of these patients may be colonized with PJP and this has been shown to exacerbate underlying lung diseases [5, 6]. However, colonization can be differentiated from infection by the presence of characteristic interstitial infiltrates, severe dyspnoea, nonproductive cough, underlying immunodeficiency, response to PJP directed treatment and absence of alternative diagnosis [7]. Concurrent COVID-19 and PJP infection has been described recently in reports from various parts of the world [8, 9 ]. A recent report described a 9.3 % *P*. *jirovecii* detection rate in critically ill COVID-19 patients [9]. This study aims to report PCR-confirmed PJP-positive cases following COVID-19 infection from a tertiary care centre in a low/middle-income country.

## Methods

Patients with PCR-confirmed PJP following COVID-19 infection who were admitted to Aga Khan University Hospital, Karachi, Pakistan from March 2020 to June 2021 were identified through a laboratory database. Detection of SARS-CoV-2 virus was performed by real-time reverse transcriptase polymerase chain reaction (RT-PCR) Cobas SARS-CoV-2 qualitative assay using Cobas 6800/8800 Systems (Roche Molecular Systems, USA). *P. jirovecii* PCR in bronchoalveolar lavage (BAL) was performed using The RealStar *Pneumocystis jirovecii* PCR kit (Altona Diagnostics GmbH). Real-time PCR amplifications were performed on a CFX96 real-time PCR detection instrument (Bio-Rad, USA). *P. jirovecii*-positive controls, an artificial heterologous extrinsic control and no-template controls were included in each run. Probes specific for the *P. jirovecii* and the internal control were labelled with the FAM and JOE fluorophores [7, 10]. Serum βeta-d-glucan (BDG) was tested using the Fungitell kit (Cape Cod, Inc., USA). Clinical parameters, radiological findings and laboratory data were obtained from electronic medical records.

## Results

During the study period, ~3707 patients were admitted with COVID-19 at our hospital. Among these, *P. jirovecii* PCR was requested in 90 patients and a positive result was identified in 10 (11 %) patients. In comparison, pneumocystis PCR data from June 2018 to March 2020 (pre-COVID-19) revealed a positivity of 15.9 % (50/314). During the study period *Pneumocystis* PCR was performed for 85 non-COVID patients and 15 (18 %) were positive. During March 2020–April 2021, a total of 85 (2.4 %) (78 pulmonary aspergillosis and 7 pulmonary mucormycosis) cases of fungal pneumonia excluding PJP were identified in COVID-19 patients.

Details for all 10 patients with post-COVID-19 PJP are provided in Table 1. The median age of the patients with PJP was 64.5 years (IQR: 50–71) and the male-to-female ratio was 7 : 3. Of the 10 PJP PCR-positive patients, 6 had diabetes mellitus and 5 had poorly controlled diabetes with HbA1C ≥6.5. Moreover, 5/10 patients had hypertension and 3/10 patients had an underlying malignancy. All patients except two had been hospitalized with severe-to-critical COVID-19; 4/10 patients had received intravenous tocilizumab for critical COVID-19. The median period between COVID-19 and the development of PJP in all patients was 29 days (IQR: 18–57). Five patients remained hospitalized with severe COVID-19 and the median period for hospitalized patients to develop PJP was 18 days (IQR: 10.5–25.5).

**Table 1.** Details of 10 patients with post-COVID-19 *Pneumocystis jirovecii* pneumonia

Case #	1	2	3	4	5	6	7	8	9	10
Age, gender	71, M	70, M	62, M	76, M	67, F	69, M	61, F	40, M	50, M	28, F
Comorbid conditions	Diabetes, hypertension, HIV status unknown	Hypertension, ischemic heart disease, Parkinson’s, BPH, HIV status unknown	Diabetes, hypertension, HIV status unknown	None, HIV status unknown	Diabetes, HIV status unknown	Diabetes, Hypertension, ischemic heart disease, chronic kidney disease, upper GI bleed, purpura fulminans, HIV status unknown	Hypertension, rheumatoid arthritis, breast cancer survivor, HIV status unknown	Diabetes mellitus, HIV status unknown	Diabetes mellitus, mantle cell lymphoma, chemotherapy (COVID pneumonia followed by PJP pneumonia 1 year back)	Acute myeloid leukaemia
Treatment for COVID	Tocilizumab, methylprednisolone	Remdesivir, tocilizumab, dexamethasone	Remdesivir, tocilizumab, dexamethasone	Methylprednisolone, remdesivir	Remdesivir, tocilizumab, dexamethasone	No treatment for COVID	Remdesivir, dexamethasone	Remdesivir, dexamethasone	Remdesivir, dexamethasone	No treatment for COVID
Course of hospitalization due to COVID	Condition improved and was discharged from hospital on day 13 with 1 week of oral systemic steroids	Condition partially stabilized, he left against medical advice on day 12 after admission on tapering doses of steroids	Course complicated with intubation at day 17. Remained admitted to hospital	Course complicated with intubation at day 14. Remained admitted to hospital	Course complicated with intubation at day 7. Remained admitted to hospital	Non-severe/ non- critical COVID with no hospitalization followed by repeated hospital admissions monthly for shortness of breath, deranged creatinine and haemodialysis	Severe COVID with need for supplemental oxygen. Condition improved and was discharged from hospital on day 6 with 1 week of oral systemic steroids	Course complicated with intubation on day 2 of admission followed by development of pneumothorax. Patient left against medical advice in critical condition	Course complicated with intubation on day 17 of admission followed by development of pneumothorax. Patient left against medical advice in critical condition	Non-severe/ non- critical COVID with no hospitalization
Duration from COVID to PJP (days)	48	56	18	23	8	180	133	18	18	36
Complaints related to PJP	Readmitted with shortness of breath and hypoxia	Readmitted with high-grade fever and shortness of breath	Worsening hypoxia	Worsening hypoxia	Worsening hypoxia	Readmitted with worsening hypoxia, respiratory distress and worsening metabolic acidosis	Readmitted with hypoxemic respiratory failure	Worsening hypoxia	Hypoxemic respiratory failure	Fever, shortness of breath and cough
Radiology	Bilateral patchy ground-glass opacities more marked in middle and lower zones. Some small cysts were also present in bilateral lower zones	Bilateral alveolar infiltrates more in mid and lower zones. Left lower lobe consolidation. Bilateral small central cysts were also present in lower zones	Bilateral diffuse ground-glass opacities, lower lobe small central cysts and consolidation, pneumomediastinum, pneumopericardium	Bilateral ground glass opacities more in lower lobes, pulmonary embolism involving subsegmental branches of bilateral pulmonary arteries	Bilateral airspace shadowing/alveolar infiltrates, more marked on right side	Bilateral non-homogenous alveolar infiltrates with bilateral mild pleural effusions	Subpleural ground-glass haziness and consolidation in bilateral lung fields along with emphysematous changes in the left upper and apical segment of left lower lobe	Bilateral non-homogenous alveolar infiltrates more in the right lower lung zone. Pneumomediastinum and subcutaneous emphysema predominantly on left side	Diffuse patchy ground-glass opacification and consolidation in bilateral lower lobes	BL lower lobe ground glass opacities that were denser at lung bases. Some small nodules and cysts also present in the same area
LDH (IU l^−1^)	297	303	410	421	445	Not performed	428	418	228	435
Lymphocyte count in the week of PJP (cells μl^−1^)	661.2	521.4	472.5	344	375	446.5	646	324	199.8	1472
Type of ventilation	Not done	Invasive	Invasive	Invasive	Invasive	Invasive	Not done	Invasive	Invasive	–
Serum BDG (pg ml^−1^)	252.3	405.9	352	395	45.621	70.682	Not done	167.885	523	<7.812
Serum GMI	0.141	0.12	0.014	3.789	0.185	0.11	Not done	1.011	0.552	0.174
BAL GMI	0.26	0.33	3.13	Not done	Not done	Not done	0.144	Not done	Not done	0.38
Other microbiological findings (infection/colonization)	* Staphylococcus aureus* in BAL (infection) BAL TB culture Xpert: negative	Not performed	*Aspergillus flavus* in BAL (infection)	Multidrug-resistant *Acinetobacter* spp., *Paecilomyces* spp. and *Hormonema* spp. in tracheal culture (infection), carbapenem-resistant *Escherichia coli* in blood (infection)	Multidrug-resistant *Acinetobacter* spp. tracheal aspirate and blood (infection)	* Stenotrophomonas maltophilia* in tracheal aspirate (colonization)	Not performed	Multidrug-resistant *Acinetobacter* spp., *Aspergillus niger*, *Aspergillus fumigatus* and *Aspergillus terreus* in tracheal culture (infection)	*A. flavus*, *A. niger* in tracheal culture (infection), positive CMV PCR (infection)	* Acinetobacter* and *Staphylococcus* species in blood culture (infection)
Antibiotics	Piperacillin/tazobactam, co-trimoxazole	Meropenem, vancomycin	Piperacillin/tazobactam, meropenem, vancomycin, co-trimoxazole	Meropenem, colistin, vancomycin, co-trimoxazole	Meropenem, colistin, vancomycin, tigecycline, co- trimoxazole	Meropenem, vancomycin, colistin, co-trimoxazole	Co-trimoxazole	Colistin, co-trimoxazole	Meropenem, colistin, co-trimoxazole	Azithromycin, meropenem, vancomycin, colistin, co-trimoxazole
Antifungals	Voriconazole (200 mg q12 h^−1^)	Amphotericin-B (50 mg q24 h^−1^)	Voriconazole (200 mg q12 h^−1^)	Voriconazole (200 mg q12 h^−1^)	Voriconazole (200 mg q12 h^−1^)	Antifungals not given	Antifungals not given	Voriconazole (200 mg q12 h^−1^)	Voriconazole (200 mg q12 h^−1^)	Amphotericin-B (40 mg q24 h^−1^)
Outcome	Discharged	Died	Died	Died	Died	Discharged	Discharged	Discharged	Discharged	Improved and discharged

BDG, beta-D-glucan; GMI, galactomannan index; TB culture, *Mycobacterium tuberculosis* culture; BPH: benign prostatic hyperplasia.

Of the 10 PJP PCR-positive patients, 6 developed acute respiratory distress syndrome (ARDS) and 4 developed pneumothorax and/or pneumomediastinum. Initial non-invasive ventilation followed by invasive mechanical ventilation and intensive care unit (ICU) admission was required by seven patients. The median BDG level was 373 (IQR: 252–406). Systemic steroids were given to eight patients and nine patients received co-trimoxazole. CT chest was performed for six patients and showed ground-glass changes consistent with both PJP and COVID-19. Of the total 10 patients, 4 patients expired, 2 patients left against medical advice in critical condition and 4 patients responded to treatment and survived. The cause of death in all four patients was ARDS, multi-organ dysfunction and septic shock. The median duration from diagnosis of COVID-19 to death or discharge was 48 days (IQR: 22–65) and from diagnosis of PJP to death or discharge it was 5 days (IQR:4–12). The trends for lymphocyte counts in all patients showed a lymphocyte count of <1000 mm^−3^ (<1.0×10^6^ cells µl^−1^) in the week of PJP diagnosis.

## Discussion

This report describes 10 patients who developed PJP after being discharged or during hospitalization with COVID-19. Five of these patients were discharged from the hospital and later presented with cough and shortness of breath and were diagnosed as having PJP. Five patients remained hospitalized with severe COVID-19 and developed PJP.

Lymphopenia is a common complication in severe COVID-19 patients that possibly led to a higher predisposition to invasive fungal infections, including PJP, in these patients. CD4^+^ T cells play a central role in counteracting *P. jirovecii* infection and an absolute lymphocyte count of ≤0.5×10^6^ cells µl^−1^ has been reported to be strongly associated with PJP in both HIV and non-HIV patient populations [4]. Additionally, the development of ARDS and the use of high-dose corticosteroids and other immunomodulatory treatment in critically ill COVID-19 patients are other contributory factors favouring PJP [1, 3]. All of our patients had a lymphocyte count of <1×10^6^ cells µl^−1^ before the diagnosis of PJP. In a recent study a low baseline lymphocyte count of ≤0.7×10^6^ cells µl^−1^ was identified as a risk factor for secondary infections [11].

All the patients reported on in this study developed PJP after a period ranging from 18 to 180 days post-SARS-CoV-2 infection. This finding is different from other reported studies so far, where PJP developed either concomitantly in COVID-19 patients or within a median duration of 10 days after ICU admission due to COVID-19 [8, 9]. However, for hospitalized patients the median duration between admission due to COVID-19 and development of PJP was 18 days. Seven out of these 10 patients did not have any prior immunosuppressive or chronic respiratory condition, while 1 was a breast cancer patient (treated) with rheumatoid arthritis on methotrexate, 1 patient had acute myeloid leukaemia and 1 patient was a known case of mantle cell lymphoma with 2 episodes of severe COVID pneumonia followed by PJP 1 year apart. Unlike the study by Alanio *et al*., where beta-d-glucan (BDG) levels were low in the majority of *Pneumocystis* PCR-positive cases, six patients in our study had raised BDG levels, confirming *P. jirovecii* infection rather than colonization [9].

Patchy areas of ground-glass opacity are the principal radiological finding in high-resolution CT scans for both PJP and COVID-19 patients. However, in PJP, a more central distribution with relative peripheral sparing is seen. In advanced cases, apart from patchy areas of ground-glass opacity [12], consolidation may be seen [13]. Lung consolidation is more common in non-HIV patients with PJP. Thin-walled cysts of varying size and shape can occur in areas of ground-glass opacity [14]. There were characteristic relatively central and lower lobe ground-glass opacities in all of our patients, with cysts observed. In COVID-19 patients, a predominantly central distribution of ground-glass opacities with cyst formation should raise suspicion of PJP and they should be evaluated for it.

Eight patients in our study received steroids, while four received tocilizumab, leading to immunosuppression and increased risk for PJP. Four patients in our series did not survive, one of these patients did not receive co-trimoxazole due to late PJP diagnosis. One patient had raised galactomannan levels in BAL with growth of *A. flavus*, and two patients had raised galactomannan levels in serum, representing a concomitant *Aspergillus* infection. Delay in diagnosis and *Aspergillus* co-infection are recognized risk factors for mortality in PJP patients [15].

PJP diagnosis is challenging in patients with COVID-19 due to similarities in presentation and problematic sampling of the lower respiratory tract. Worsening hypoxemia may be a long-term COVID-19 sequelae and would require a high index of suspicion from the physician to include investigation on the lines of PJP in addition to other infections such as tuberculosis or cytomegalovirus (CMV). PJP laboratory diagnosis is either immunofluorescence- or PCR-based, both performed on bronchoalveolar lavage samples for maximal sensitivity. COVID-19 patients are difficult to sample due to worsening hypoxemia. Even though deep tracheal secretions have been validated for PJP testing in our laboratory, tenacious secretions can be difficult to process. False positivity can be an issue in such cases when using superficial secretions in colonized patients: quantitative PCR may help but will need optimization and add cost [16]. Positive panfungal antigen BDG in serum is nonspecific and may indicate candidaemia or COVID-19-associated pulmonary aspergillosis (CAPA), and sensitivity in COVID-19 patients may be low [17, 18].

In our study, we distinguished infection from colonization based on the following. The majority of patients developed PJP after a median duration of approximately 1 month. Among those who survived, they had recovered from their initial illness due to COVID-19 and were readmitted after a month with new symptoms of hypoxia and imaging features and BAL findings consistent with PJP and were treated with trimethoprim sulfamethoxazole with clinical improvement. Among non-survivors, most had a delayed presentation with COVID-19, had a prolonged hospital course with refractory hypoxia and new development of pneumothorax and pneumomediastinum, prompting testing for PJP and subsequent diagnosis.

As mentioned, elevated BDG was present in all patients, while GM was not positive even in those who had *Aspergillus* spp., which has greater specificity for CAPA. CAPA coinfection with PJP in COVID-19 patients has been reported in the literature [18]. However, we recognize that the distinction between infection and colonization with *P. jirovecii* is challenging and not being able to perform qPCR or microscopy is a limitation of our study.

In summary, invasive fungal infections such as PJP should be considered as a complication in COVID-19 patients, with prompt evaluation and management.
